# Biophysical Detection of Diversity and Bias in GPCR Function

**DOI:** 10.3389/fendo.2014.00026

**Published:** 2014-03-05

**Authors:** Werner C. Jaeger, Stephen P. Armstrong, Stephen J. Hill, Kevin D. G. Pfleger

**Affiliations:** ^1^Molecular Endocrinology and Pharmacology, Harry Perkins Institute of Medical Research and Centre for Medical Research, The University of Western Australia, Perth, WA, Australia; ^2^Cell Signalling Research Group, School of Life Sciences, Queen’s Medical Centre, University of Nottingham Medical School, Nottingham, UK; ^3^Dimerix Bioscience Pty Ltd, Perth, WA, Australia

**Keywords:** bioluminescence resonance energy transfer, BRET, fluorescence resonance energy transfer, FRET, GPCR, GPCR-HIT, heteromer, Receptor-HIT

## Abstract

Guanine nucleotide binding protein (G protein)-coupled receptors (GPCRs) function in complexes with a range of molecules and proteins including ligands, G proteins, arrestins, ubiquitin, and other receptors. Elements of these complexes may interact constitutively or dynamically, dependent upon factors such as ligand binding, phosphorylation, and dephosphorylation. They may also be allosterically modulated by other proteins in a manner that changes temporally and spatially within the cell. Elucidating how these complexes function has been greatly enhanced by biophysical technologies that are able to monitor proximity and/or binding, often in real time and in live cells. These include resonance energy transfer approaches such as bioluminescence resonance energy transfer (BRET) and fluorescence resonance energy transfer (FRET). Furthermore, the use of fluorescent ligands has enabled novel insights into allosteric interactions between GPCRs. Consequently, biophysical approaches are helping to unlock the amazing diversity and bias in G protein-coupled receptor signaling.

## Introduction

Guanine nucleotide binding protein (G protein)-coupled receptors (GPCRs), also known as seven-transmembrane receptors, form the largest gene family in the vertebrate genome, with 1000–2000 members (1). To maintain homeostasis, GPCRs mediate responses to a vast array of extracellular signals, including light, ions, odorants, nucleotides, amino acids, glycoproteins, proteases, neurotransmitters, peptide hormones, lipids, and mechanical energy. Accordingly, GPCRs participate in many physiological and pathological processes, and the ability of their function to be modified with compounds presents GPCRs as highly useful pharmaceutical targets. Indeed, it has been widely reported that between 30 and 50% of medicines target this class of receptor (2–4). However, a recent study reported that these drugs only target approximately 30% of known non-olfactory GPCRs, and a much smaller percentage of potential underlying targets have been identified (5). The advent of increasingly powerful techniques to investigate GPCR function has progressed the field from a classical bimodal, monomeric theory of GPCR activity to involve multimodal, oligomeric mechanisms. Multiple alternative signaling pathways, allosterism, ligand bias, and receptor–receptor interactions all generate diverse GPCR trafficking and signaling outcomes. Notably, these mechanisms are not independent of each other, but instead likely integrate to influence drug specificity and efficacy. They are therefore the subject of intense focus for ongoing and future drug development.

## Diversity of GPCR Function in Oligomeric Complexes

Guanine nucleotide binding protein (G protein)-coupled receptors were formerly believed to function as monomeric entities, although data from a number of early studies suggested otherwise [reviewed in Ref. (6–8)]. Most notably, early work involving the seminal GABA_B1_/GABA_B2_ heteromer has lent support to the concept of GPCRs acting as oligomeric complexes (9–11), along with studies of the taste receptor family (12–14). Biochemical and increasingly powerful biophysical methods have helped strengthen the growing body of evidence supporting the ability of GPCRs to form such complexes (15–21), including homomers, heteromers, and higher-order quaternary complexes (8, 22–25). When present in oligomeric complexes, individual receptors (protomers) may differ in their ability to bind ligands, traffic to/from the cell surface, and modify overall signaling activity compared to when present individually or in alternative complexes. In this way, individual receptors within a macromolecular complex provide a level of allosteric modulation that can ultimately alter the trafficking and signaling output of the complex as a whole. Furthermore, while these activities may be categorized and discussed as distinct events, allosteric modulation of ligand binding, signaling, and trafficking of receptors through oligomerization are highly dependent processes.

## Recent Advances in Measurement of GPCR Complexes Using Biophysical Techniques

Recent advances in biophysical methods to monitor protein–protein interactions have evolved through the discovery and optimization of novel luciferase enzymes/substrates and fluorophores, as well as improved sensitivity of detection instrumentation. Variants of green fluorescent protein (GFP) derived from *Aequorea victoria* have resulted in optimization of the chemical stability as well as the quantum yield of these proteins, enabling them to be used to measure protein–protein interactions with greater sensitivity, as well as for use in living organisms (26). Novel luciferases, such as the optimized *Renilla* luciferases (26, 27) and newly characterized enzymes from other marine species (28), are providing molecular endocrinologists and pharmacologists with more optimal tools to assess complex formation and function utilizing resonance energy transfer (RET) techniques *in vitro* and *in vivo*. These assays utilize the biophysical properties of Förster RET that involves non-radiative transfer of energy from an excited donor to a suitable acceptor. Fluorescence resonance energy transfer (FRET) utilizes a donor fluorophore (such as cyan fluorescent protein) that is excited by an external light source (such as a flash lamp or laser), whereas bioluminescence resonance energy transfer (BRET) utilizes a luciferase enzyme as donor that transfers energy upon oxidation of its substrate. These approaches therefore enable live cell protein proximity detection (29–31) and can be used to monitor changes in conformations of individual GPCR protomers as well as between multiple GPCRs in complexes.

### Fluorescence resonance energy transfer/time-resolved FRET

A number of FRET-based techniques exist and have been extensively reviewed elsewhere (32). Intensity-based FRET methods have their limitations due to the overlap of fluorophore excitation and emission spectra (33). Bleedthrough of the donor emission into the acceptor detection window needs to be removed, as does the component of the acceptor emission resulting from direct acceptor excitation. Spectral unmixing is one approach to address this issue (34).

Alternatively, the “unmixing” can occur temporally through the utilization of time-resolved FRET (TR-FRET), which can also overcome issues of photobleaching and high background autofluorescence (35). This approach relies on energy transfer between lanthanide donors (such as europium or terbium cryptate complexes) and a suitable acceptor (such as the Cy-5-like cyanine dye, d2, or the modified allophycocyanine, XL665). The exceptional duration of fluorescence emission (300–1000 μs) from lanthanide donors enables measurement of acceptor emission long after background fluorescence has decayed (35). This, together with the high FRET efficiency between lanthanide and cyanine fluorophores, and the chemically stable nature of cryptate complexes, results in excellent signal-to-noise ratios for TR-FRET. As such, TR-FRET assays are well suited to high-throughput screening (36), as well as general investigative studies. When combined with labeling methods such as SNAP- and CLIP-tag technology or fluorescent ligands, TR-FRET becomes a powerful tool for detecting GPCR proximity. For example, a recent paper from the Milligan laboratory investigated cannabinoid receptor 1 (CB_1_) and orexin receptor 1 (OX_1_) heteromerization using TR-FRET and SNAP/CLIP tags, providing evidence for a remarkable ability of OX_1_ to induce internalization of the CB_1_–OX_1_ heteromer with greater potency than observed with OX_1_ alone (37). Similar approaches have been used to determine the stoichiometry of homo- and heteromer populations at the cell surface, specifically within the metabotropic glutamate (mGlu) receptor family (38) or between dopamine D2 and D3 receptors (39).

Fluorescent ligands (see section below) offer a powerful alternative approach to labeling GPCR complexes (40–42), by removing the requirement for engineered fusion proteins. Consequently, they enable investigation of GPCR complexes in their native environment, which is particularly useful given that the existence of heteromers *in vivo* is much less well-established than *in vitro*. Notably, TR-FRET between fluorophore-conjugated antagonists has been used to demonstrate the existence of oxytocin receptor homomers in primary mammary tissue (43, 44).

A recent paper demonstrated the use of TR-FRET antibodies to quantify levels of EGFR/HER2 heterodimerization since simultaneous *in vivo* treatment with antibodies that disrupted this complex resulted in the greatest median survival rate (45). Such a technique could also be used for screening compounds that regulate heteromerization of GPCR complexes, depending upon the availability of suitably validated GPCR antibodies and a distinct and measurable functional effect of a particular complex. The presence and proximity of protomers that constitute a GPCR complex may also be inferred using epitope-tagged receptors and fluorescently labeled antibodies to these epitopes. For example, to detect proximity between CXCR3 and CXCR4, these receptors were N-terminally epitope-labeled (HA-CXCR3/FLAG-CXCR4) and TR-FRET was measured between TR-FRET-labeled antibodies to these epitopes (46).

### Bioluminescence resonance energy transfer

Following the oxidation of a suitable substrate by a luciferase enzyme, typically a variant of *Renilla* luciferase (Rluc), such as Rluc2 or Rluc8, BRET occurs through a non-radiative transfer of energy to a complementary fluorophore, such as a variant of GFP, if it is in sufficiently close proximity (47, 48). Critically, as with FRET, energy transfer is dependent upon the distance between donor and acceptor (inversely proportional to the sixth power), as well as their relative orientation and degree of spectral overlap (29, 30, 49). The BRET process occurs naturally in marine organisms such as the jellyfish *Aequorea victoria* and sea pansy *Renilla reniformis*. Since the seminal use of this technique to observe interacting clock proteins (50), BRET has been used increasingly to monitor proximity indicative of association, dissociation, or conformational changes involving proteins of interest (29). Indeed, this method can be used to observe intramolecular changes to protein conformation in a bimodal manner indicating active or inactive states. This is particularly important for biosensors, and will be detailed in the next section (see Resonance Energy Transfer Biosensors).

Recent innovations in BRET technology have been extensively reviewed (30), but briefly, the original BRET approach (now termed BRET^1^) generally uses an Rluc variant and yellow fluorescent protein (YFP), as the donor and acceptor respectively, with coelenterazine h as the enzyme substrate. A second generation, termed BRET^2^, uses a synthetic coelenterazine, DeepBlueC, with a blue-shifted donor emission spectrum, and modified GFP (GFP^2^) (51). GFP10 is also a suitable BRET^2^ acceptor (52–55). BRET^2^ has the advantage of increased spectral resolution between donor and emission peaks, but this is overshadowed by rapid decay kinetics of the substrate and a substantially reduced quantum yield (56, 57). The distance ranges over which BRET^1^ and BRET^2^ energy transfer occurs are comparable with FRET, with BRET^1^ being more sensitive to proximity than BRET^2^ (58). BRET^1^ with Rluc/enhanced YFP, and BRET^1^ with Rluc2 or Rluc8/Venus, exhibit minimal energy transfer beyond about 6.5 and 8 nm, respectively, whereas BRET^2^ extends to about 11 nm regardless of whether Rluc, Rluc2, or Rluc8 is used (58).

Another permutation of BRET known as “extended BRET” (eBRET) utilizes a protected variant of coelenterazine h (EnduRen) and enables real-time monitoring of interactions for extended time periods (47, 59, 60). This enables discrimination of distinct kinetic profiles, such as between mutated receptors (61, 62). Similarly, protected DeepBlueC variants have also been produced, and may be of use to take advantage of increased spectral resolution with a lengthened substrate half-life (63). A further modification known as BRET^3^, utilizing a mutant fluorescent protein with red-shifted emission (mOrange), has also proved useful for *in vivo* imaging (64, 65). Other variations of BRET, including multiplexing, have also been devised to allow multiple acceptor fluorophores, conjugated to various proteins of interest, to be activated in a cascade manner (66). For example, this has been used to detect proximity between three proteins in a GPCR complex (67).

Recently, an investigation was carried out into novel luciferases from marine organisms that resulted in the isolation and optimization of a novel luciferase, “NanoLuc™,” derived from the deep sea shrimp *Oplophorus gracilirostris* (28). Concurrently, a novel imidazopyrazinone substrate, furimazine, was specifically developed for use with this novel enzyme. NanoLuc™ is almost half the size of *Renilla* luciferase [19 versus 36 kDa, respectively (28)], and this could potentially result in a lower degree of interference or steric hindrance of the luciferase tag when fused to a protein of interest in mammalian cells. NanoBRET™ benefits from the substantially increased brightness of NanoLuc™(28), enabling very low levels of enzyme expression to be used. Furthermore, the 460-nm emission maximum of NanoLuc™(28) is blue-shifted compared to the Rluc variants with emission peaks of about 480 nm (57). Along with the approximately 20% narrower spectral emission of NanoLuc™(28), this enables better spectral separation from the acceptor emission.

### Resonance energy transfer biosensors

Biosensors are novel molecular tools that can be used to investigate the activity of a signaling or structural protein qualitatively and/or quantitatively (68, 69). This may be in the form of bimodal output, whereby either unimolecular or bimolecular peptide probes are synthesized to include certain domains that measure a change in the inactive or active state of an effector protein, and this consequently causes a change in conformation of the biosensor, and magnitude of signal output (68, 69). Importantly, these can be used as screening tools to measure the activity of signaling or structural protein activity in a high-throughput situation. Several FRET biosensors have been developed for small GTPases, involved in a number of regulatory and signaling activities in the cell (68). Intracellular Ras activity of angiotensin receptor type 1 (AT_1_R) stimulated by angiotensin II has been observed using a Ras biosensor, and found to have activity in certain compartments (70). GTPases are activated and deactivated by guanine nucleotide exchange factors (GEFs) and GTPase activating proteins (GAPs), respectively (71). Therefore, if these proteins are known, and the domains that they affect, fusion proteins can be developed involving either a single domain, or two domains. If the effector causes a considerable change in conformation, this may be sufficient to enable a difference in the RET signal to be detected. This has been developed for Rab5, a member of the Rab family of GTPases involved in trafficking of GPCRs and other proteins in transport vesicles throughout various compartments in the cell (71), Quantitative changes using FRET probes have typically been used as the RET method for a large proportion of characterized biosensors, however, BRET probes are increasingly being used. An ERK sensor “REV” has been developed incorporating BRET tags to measure the phosphorylated or unphosphorylated state of ERK (72). Similarly, G protein activation can be inferred by monitoring interactions between G proteins and GPCRs using BRET (53, 73–75). Other examples include BRET biosensors for cAMP (76) and protein kinase A (77) [see Ref. (78) for review]. Although biosensors can be created using artificial constructs, native proteins may also be tagged at either N- or C-termini, or by integrating FRET or BRET tags into internal domains of the protein, such as the third intracellular loop of GPCRs, as recently demonstrated for an odorant GPCR (79). Additionally, homogeneous time-resolved fluorescence (HTRF) is a biosensor platform with a range of applications (80), including measurement of inositol-1-phosphate (61, 81, 82) and cAMP (61). Due to the impermeability of the metal chelates, HTRF measurement either requires cell lysis or mild cell permeabilization, the latter with Triton X-100 for example (75).

Proximity between BRET-tagged β-arrestin and BRET-tagged ubiquitin can be monitored following activation of a co-expressed GPCR, and the resultant kinetic profiles provide interesting insights into receptor pharmacology, as seen when comparing V_2_R with β_2_ adrenoceptor (83) or different orexin receptor subtypes (62, 84). Alternatively, BRET^1^ and BRET^2^ can be utilized in parallel by co-expressing Rluc-tagged β-arrestin, YFP-tagged GPCR, and GFP^2^-tagged ubiquitin, then measuring BRET in parallel cell populations following addition of coelenterazine h (BRET^1^) or DeepBlueC (BRET^2^) substrate (83).

### Detection and profiling of GPCR heteromer complexes

The agreed definition of a receptor heteromer is a “macromolecular complex composed of at least two (functional) receptor units with biochemical properties that are demonstrably different from those of its individual components” (85). Notably, heteromeric complexes may exist without GPCRs interacting directly, as other complex components may be in between (86). To detect specific heteromer complexes of GPCRs, suitable experimental controls are required to differentiate between specific and non-specific (bystander) reporter signals.

**Figure 1:**
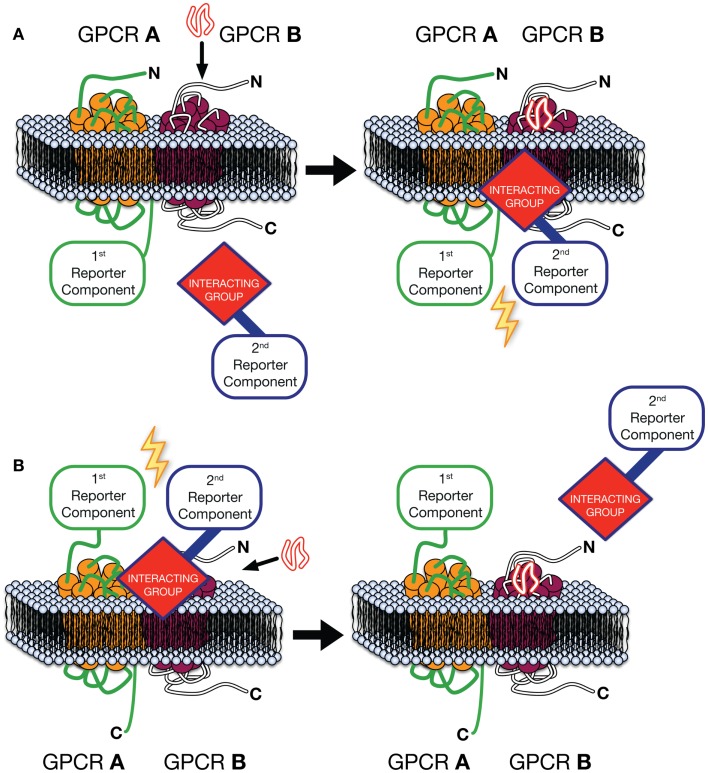
**GPCR-Heteromer Identification Technology (GPCR-HIT)**. GPCR A is fused to the first reporter component, GPCR B is unlabeled with respect to the reporter system, and a GPCR-interacting group is linked to the complementary second reporter component. The first reporter component is fused to the C-terminus of the GPCR and an intracellular interacting group is used **(A)**, or ligand binding to the heteromer is assessed by fusing the first reporter component to the N-terminus of a GPCR and using a fluorescently labeled ligand as the interacting group/second reporter component combination **(B)**.

The Receptor-Heteromer Investigation Technology (Receptor-HIT) (87), includes Receptor Tyrosine Kinase-HIT (RTK-HIT) (88) and the GPCR-Heteromer Identification Technology (GPCR-HIT) as a novel approach for rapid identification, screening, and profiling of GPCR heteromers (30, 46, 86, 87, 89–92) (Figure 1). The approach consists of three essential components co-expressed in live cells, (i) a GPCR fused to a proximity-based first reporter component, (ii) an unlabeled GPCR, and (iii) a GPCR-interacting group, linked to the complementary second reporter component, whose interaction with the complex is modulated upon binding a ligand selective for the unlabeled GPCR or the heteromer complex specifically. Typically the first reporter component is fused to the C-terminus of the GPCR and an intracellular interacting group is used (Figure 1A), such as β-arrestin (86) or tagged G protein subunits (75). Indeed, our work with EGFR–HER3 complexes recruiting Grb2 illustrates the diversity of potential intracellular receptor interacting partners (88). However, by fusing the first reporter component to the N-terminus of a GPCR and using a fluorescently labeled ligand as the interacting group/second reporter component combination (Figure 1B), GPCR-HIT can be used to assess ligand binding to the heteromer. Upon expression of the aforementioned three components in cells, a ligand specific for the unlabeled GPCR or the heteromer complex is added and the reporter signal measured. If the GPCRs are not proximal, addition of the ligand specific to the untagged GPCR will modulate the interacting group’s proximity to the activated receptor, however, as the two reporter components will not be in close proximity, no modulation of the reporter signal will be measured. In contrast, when heteromers of the GPCRs are present, addition of ligand will modulate the interacting group’s proximity to the complex, resulting in a change in reporter signal (Figure 1).

GPCR-HIT can be used on a broad range of reporter assay platforms including FRET (e.g., CisBio’s HTRF^®^), BRET, bimolecular fluorescence complementation (BiFC), bimolecular luminescence complementation (BiLC), enzyme fragment complementation (e.g., DiscoveRx’s PathHunter^®^), and proteolysis-based reporter systems (e.g., Invitrogen’s Tango™). The GPCR-HIT technique was recently demonstrated for two well characterized heteromers, CCR2–CXCR4 and CCR2–CCR5 (86). In this study, untagged CCR2 was co-expressed with β-arrestin2/Venus YFP and Rluc8-tagged CXCR4 or CCR5. Addition of CCL2, the ligand selective for CCR2, caused an increase in BRET signal indicative of β-arrestin recruitment to the heteromeric complex (86). In addition to dose–response and kinetic profiles, *Z*′ factors were determined (>0.6) for β-arrestin recruitment, which demonstrate the suitability of this assay for drug screening programs (86).

GPCR-Heteromer Identification Technology was used to identify and profile a novel α_1A_AR–CXCR2 heteromer, potentially relevant to benign prostate hyperplasia (BPH) (92). The α_1A_AR is an important mediator of prostatic smooth muscle tone and lower urinary tract function. Consequently, α_1A_AR antagonists are used in the treatment of BPH (93). Typically, α_1A_AR interacts extremely weakly, if at all, with β-arrestin in HEK293 cells (94). However, α_1A_AR coimmunoprecipitates with β-arrestin in prostate smooth muscle cells (95), suggesting a necessary cofactor may be missing in HEK293 cells. We employed GPCR-HIT in HEK293 cells using BRET with β-arrestin2/Venus as the acceptor. When α_1A_AR/Rluc8 was expressed alone, norepinephrine failed to induce recruitment of β-arrestin2/Venus to the receptor. However, when CXCR2 was co-expressed, norepinephrine caused a marked increase in BRET signal, suggestive of a heteromeric complex. Further investigation using CXCR2/Rluc8 and untagged α_1A_AR with β-arrestin2/Venus revealed a much larger increase in BRET signal compared to the reverse (BRET tag) configuration, indicating that β-arrestin2 may be recruited to CXCR2 via an allosteric interaction with norepinephrine-activated α_1A_AR. Interestingly, the norepinephrine-dependent β-arrestin recruitment was inhibited by SB265610, a CXCR2-specific inverse agonist, in addition to Terazosin, an α_1A_AR antagonist. Furthermore, BRET studies with both receptors tagged suggest that α_1A_AR–CXCR2 heteromerization is constitutive and not ligand-dependent (92).

Critically, the increase in signal observed with GPCR-HIT is ligand-dependent and specific to the heteromeric complex. The latter attribute is particularly important for demonstrating unique pharmacology arising from heteromerization, whilst the former enables heteromer-specific or biased compound screening and profiling (89, 91). BRET saturation and competition assays are often used to demonstrate the specificity of an interaction, however, they do not provide important functional information, as demonstrated when BRET saturation experiments were used to assess the α_1A_AR–CXCR2 heteromer using α_1A_AR and vasopressin receptor 2 (V_2_R) as a control (92). Surprisingly, co-expression of α_1A_AR and V_2_R also resulted in a hyperbolic curve indicative of specific proximity, despite a distinct lack of noticeable change in receptor pharmacology and an absence of norepinephrine-induced β-arrestin recruitment. In contrast, norepinephrine caused a marked increase in β-arrestin recruitment to the α_1A_AR–CXCR2 heteromer when compared to α_1A_AR alone (92). BRET competition assay data, where increasing expression of an unlabeled receptor reduces the BRET signal between BRET-tagged receptors, should also be interpreted with caution. This is because increasing the expression of unlabeled receptor can result in lower expression of the BRET-tagged receptors, artifactually resulting in a lower BRET signal (29). Therefore, whenever such competition data are presented, they should be supported by data showing relative receptor expression levels in the presence and absence of the competitor.

### Protein-fragment complementation assay

**Figure 2:**
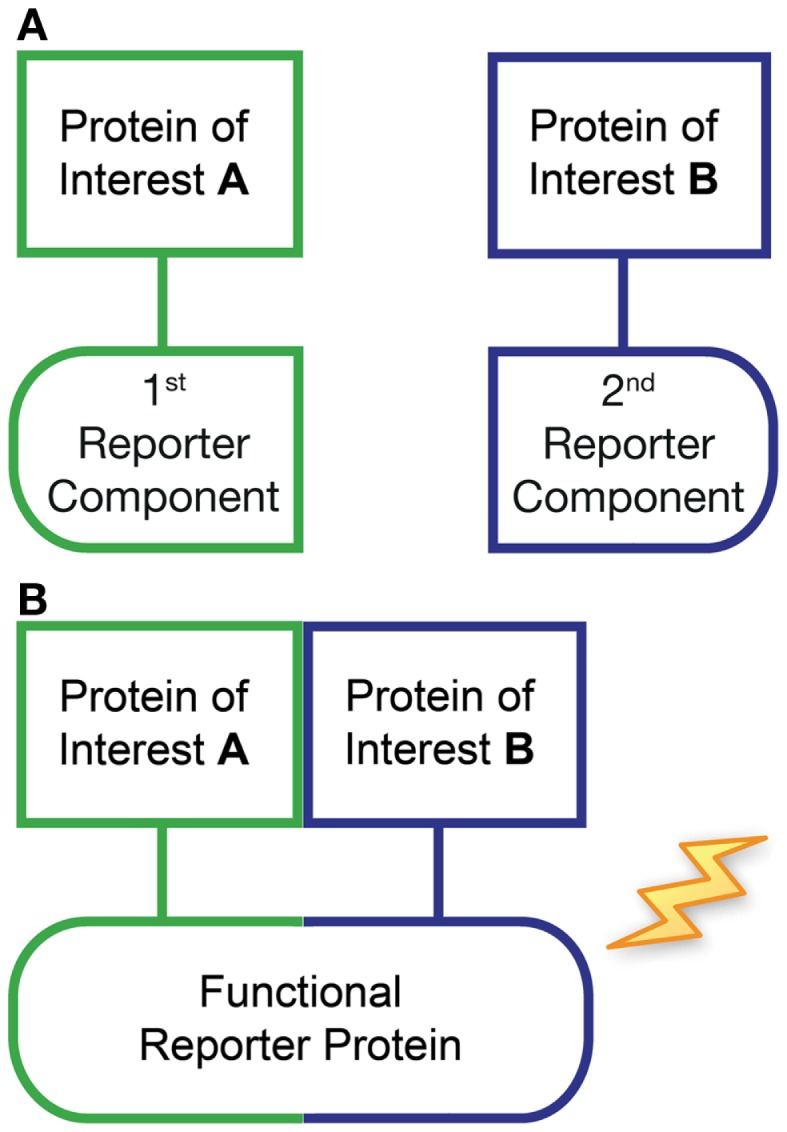
**Protein-fragment complementation assay (PCA)**. This approach typically utilizes the expression of two recombinant proteins of interest fused to fragments of a split reporter protein (first and second reporter components). In the absence of any interaction between the proteins of interest, the separate reporter fragments remain inactive **(A)**. However, if the two proteins of interest come into close proximity then the accompanying complementary reporter fragments are capable of recombining to form a functional protein, resulting in an increase in reporter signal **(B)**.

Protein-fragment complementation assay (PCA) represents another useful method for examining protein–protein interactions both *in vitro* and *in vivo* (96, 97). With this approach, each protein of interest is fused to one component of a split reporter protein (Figure 2). In the absence of any interaction, the separate fragments remain inactive (Figure 2A), however, when the two proteins of interest interact in an appropriate manner, the complementary fragments recombine to form a functional reporter protein (Figure 2B). Various reporters can be used including β-lactamase, dihydrofolate reductase, tobacco etch virus (TEV) protease, GFP variants (termed BiFC), and luciferase (termed bimolecular luminescence complementation, BiLC) (98, 99). Each has particular advantages and limitations depending on the application. For example, BiFC can be combined with fluorescence microscopy to investigate the intracellular localization of the interacting proteins. However, the recombination of GFP variants is irreversible, and interactions cannot be visualized in real time due to the slow maturation of the GFP. In contrast, luciferase-based PCA fragments appear to mature faster and are thought to be more reversible (97, 100). On the other hand, luminescence-based assays are generally not suitable for high resolution imaging. PCA is particularly useful for detecting weak protein–protein interactions and can provide some measure of affinity, as for weak interactions, BiFC signals are believed to be proportional to interaction strength (101). However, careful selection of experimental controls is also required (99), as by necessity the two reporter fragments must retain some affinity for one another. Nevertheless, when combined with other approaches, PCA is clearly a powerful technique. For example, BiFC and GPCR-HIT were recently used in parallel to investigate the novel pharmacology of AT_1_–AT_2_ heteromers (90).

### Combinations of PCA and RET approaches

Protein-fragment complementation assay can also be combined with BRET or FRET to examine complexes containing three or more proteins. For example a combination of BRET and BiFC, using Rluc-tagged adenosine A_2A_ receptors (A_2A_R), along with A_2A_R-N-YFP and A_2A_R-C-YFP, was used to detect higher-order oligomeric complexes of A_2A_R (102). Similarly, BRET experiments have been carried out with dopamine D_1_ and D_2_ receptors tagged with split Rluc8 fragments as well as Venus-tagged Gα subunits, to demonstrate functional selectivity arising from heteromerization (103). Furthermore, by using four fragments (split Rluc and split Venus), Guo et al. demonstrated that the dopamine D_2_ receptor (D_2_R) can exist in a complex of at least four receptor protomers (104). BRET has also been combined with FRET to give sequential RET (SRET) (105). Combinations of three fusion proteins were used, Rluc-, YFP-, and DsRed-tagged (SRET^1^) or Rluc-, GFP^2^-, and YFP-tagged (SRET^2^) (105). If all three proteins are present in a complex, then Rluc substrate oxidation enables energy transfer from Rluc to the BRET acceptor (e.g., YFP), which then has the potential to act as a FRET donor to give sequential energy transfer to the FRET acceptor (e.g., DsRed). Complex formation is then inferred by specific measurement of FRET acceptor emission. This approach was recently used to provide evidence for heteromerization of adenosine A_2A_, dopamine D_2_, and cannabinoid CB_1_ receptors (105). Similarly, SRET was used to investigate higher-order complexes of adenosine A_2A_, D_2_, and mGlu_5_ receptors (106).

### Proximity ligation assay

Proximity ligation assay (PLA) is a highly sensitive technique used to directly visualize protein–protein interactions with single-molecule resolution (107). PLA is an antibody-based approach of which several variations exist [see Ref. (108) for review]. In one of the more commonly used formats, two proteins of interest are targeted with primary antibodies from different species, and further labeled with two specific secondary antibodies conjugated to oligonucleotides. Complementary oligonucleotides are added to enable proximity-dependent (<40 nm) hybridization and ligation to form a circular DNA template. The template is then amplified *in situ* and visualized with a fluorescently labeled oligonucleotide probe. This approach yields a number of advantages over well-established imaging techniques in that the amplification step enables the visualization of individual protein complexes, whilst the *in situ* nature of the assay allows for determination of subcellular localization. Furthermore, as bioengineered protein constructs are not required, PLA can be used to visualize protein–protein interactions in primary tissue. Notably, PLA was used in a recent study in the Javitch laboratory to identify dopamine D_2_ and adenosine A_2A_ receptor heteromers in the striatum of mice *ex vivo* (109). An important caveat is that PLA requires the use of well-validated antibodies, which are not always available for GPCRs. Nevertheless, PLA remains a powerful technique for investigating GPCR heteromers, as exemplified by studies on dopamine D_2_–D_4_ receptor heteromers in HEK293T cells (110) and cysteinyl leukotriene receptor-1 and -2 heteromers in INT-407 intestinal epithelial cells (111).

## Fluorescently Labeled Ligands to Detect GPCR Complexes

The cellular context within which a receptor is located can have a major impact on ligand-binding affinity, efficacy, and the signaling pathways that are subsequently activated (112–114). It is therefore important to derive methods for the measurement of ligand-binding affinity in living cells where the integrity of the local cellular environment is maintained under physiological conditions. Fluorescence-based ligand-binding assays have the sensitivity and resolution to make measurements at the single-cell level and high quality fluorescent ligands (both agonists and antagonists) have become available in recent years to study GPCRs (115–119). These have been successfully applied to imaging experiments using confocal microscopy (117, 118, 120–122) and more recently to fragment screening strategies in living cells using automated confocal imaging plate readers (123). The ability to monitor ligand binding with fluorescent ligands in real time and at the level of single living cells has also provided powerful insights into the kinetics of ligand association and dissociation (42, 124, 125). Furthermore, the ability to evaluate the influence of non-fluorescent ligands on the dissociation kinetics of an orthosteric fluorescent ligand has provided an opportunity for the study of allosterism and negative cooperativity across dimer interfaces (42, 124, 125).

Fluorescent ligands have also been utilized with total internal reflection fluorescence (TIRF) microscopy. For example, a recent study identified individual M1 muscarinic receptors and determined their dimerization kinetics using TIRF and the fluorescent antagonist, Cy3B–telenzepine (126). Similarly, the monomer/dimer equilibrium of *N*-formyl peptide receptors was measured using TIRF and Alexa Fluor 594-conjugated *N*-formyl hexa-amino-acid peptide (127).

In addition to the direct imaging of the binding of a fluorescent ligand to an untagged wild-type receptor, fluorescent ligands have also been used in TR-FRET applications where the ligand and receptor (N-terminal) have been labeled with compatible TR-FRET partners (41). This has also been adapted to apply TR-FRET to the study of receptor dimers in native tissues (43). A similar strategy can be applied with NanoBRET™, using fluorescent ligands and GPCRs tagged on their N-terminus with NanoLuc™.

### Fluorescence correlation spectroscopy and the measurement of ligand-binding in single cells, membrane microdomains, and nanodiscs

Biased signaling to particular intracellular signaling pathways (113, 128–130) can be considered to be equivalent to the regulation of GPCR ligand-binding and efficacy caused by small allosteric ligands. In this case, however, it is the binding of intracellular signaling proteins to the intracellular facing domains of the GPCR that mediates the allosteric effect. It is well-established that allosteric ligands bind to a site distinct from the orthosteric binding site occupied by the endogenous natural ligand and produce conformational changes in the receptor that may alter orthosteric ligand-binding affinity, agonist efficacy, or both (113, 131–133). In a similar manner, the interaction of a signaling protein with a receptor has the potential to change the binding affinity of the orthosteric ligand as a consequence of this allosteric interaction. A feature of allosterism is that the effects observed are probe-dependent (113, 132, 133). This means that the consequence of any allosteric influence on the receptor will depend on the ligand (probe) occupying the orthosteric site. As a consequence, agonists will usually have a higher affinity for the receptor when it is bound to an intracellular signaling protein and this will depend on both the orthosteric agonist involved and the signaling protein to which the receptor is coupled. This can manifest as signaling bias.

The location of the receptor and the signaling proteins can also have a major impact on the signaling outcome of receptor activation. It is now known that GPCRs and signaling proteins can be compartmentalized within the cell membrane (134–137). It is also clear that signaling from GPCRs can be mediated from intracellular domains following their internalization (138–141). This not only provides a mechanism by which intracellular signaling can be orchestrated by location within a cell, but also points to the potential for different signaling pathways to be activated by the same receptor in different cellular or membrane locations (135–137). The latter possibility raises the need to develop techniques by which the pharmacology of a receptor in a specific domain or location can be monitored. One such technique is fluorescence correlation spectroscopy (FCS) (142).

Fluorescence correlation spectroscopy is a quantitative biophysical technique that can measure the diffusional characteristics and estimate the number of fluorescent particles (e.g., GPCR complexes, signaling proteins, or fluorescent ligand-occupied receptor complexes) within highly localized membrane microdomains of single living cells (~0.2 μm^2^) (142–146). Fluorescent ligands in combination with FCS have also been used to study the properties and behavior of a number of different GPCRs in discrete membrane microdomains of single cells (117, 120, 134).

Fluorescence correlation spectroscopy uses a small confocal detection volume created by focusing a laser to a diffraction-limited spot using a lens with a high numerical aperture [see Ref. (142) for further details]. Essentially, the resulting detection volume is approximately 0.25–0.5 fl depending on the particular excitation wavelength being used (i.e., a larger volume is created with a red 633 nm laser compared to a green 488 nm laser). As fluorescent molecules (free fluorescent ligands, receptor-bound fluorescent ligands, GFP-tagged receptor complexes) diffuse through this volume, they are excited by the laser and the emitted photons are detected in a time-correlated manner using a single-photon counting device (e.g., an avalanche photodiode). Over a period of time, this leads to fluctuations in the measured mean level of fluorescence intensity. Autocorrelation analysis (142) of these fluctuations provides information on how long the species responsible for a particular fluorescent fluctuation is present within the confocal volume (the average dwell time, *t*_D_) and also the number (*N*) of diffusing fluorescent species present. As the fluorescent fluctuation data are collected in a time-correlated manner, it is possible to extract from this information the number of fluorescent particles present with particular *t*_D_ values in complex mixtures (142).

In the context of ligand-binding experiments, it is therefore possible to obtain from FCS experiments the concentrations (deduced from *N* and the actual confocal volume) of both fluorescent ligand and ligand–receptor complexes on the basis of their differing diffusion coefficients (117, 120, 134, 142). For example, previous FCS work with a fluorescent adenosine A_3_ receptor (A_3_R) agonist in CHO cells expressing the human A_3_R revealed an agonist-occupied A_3_R complex with a membrane diffusion coefficient of 0.12 μ^2^m/s (134). It is worth pointing out that this is too slow to represent a single receptor and is more likely to be representative of an oligomeric signaling complex within which the receptor resides (117). Competition studies with an A_3_R-antagonist (MRS1220) and an A_3_R-agonist (NECA) indicated that this component had high affinity (low nanomoles) for both agonists and antagonists (134). These data suggest that, at the very low concentrations of fluorescent A_3_R-agonist required for FCS, selective labeling of the active (R*) form of the A_3_R can be achieved (134). Furthermore, the lack of effect of pertussis toxin on this high-affinity agonist binding suggested that the agonist-occupied receptor detected was not coupled to G_i_ proteins (134).

The ability of FCS to effectively work at high resolution in single-photon detection mode means that it also has the potential to monitor ligand-binding events to solubilized and purified GPCRs. The most successful approach to the reconstitution of these purified GPCRs into a membrane environment has come from the use of high-density lipoproteins (HDLs). HDLs, which are composed of a dimer of apolipoprotein A–I surrounding a planar bilayer of approximately 160 phospholipids, can be reconstituted *in vitro* to produce a disk-shaped structure (nanodisc or nanolipoprotein particle) of 10–12 nm in diameter and a thickness of 40 Å (147). Recently, nanodiscs have been used to solubilize the Neurokinin 1 receptor (NK1R) and to investigate the kinetics of the binding of fluorescent substance P to substance P-bound NK1R contained within nanodiscs (148). The combination of these techniques has the potential to investigate ligand-binding to defined receptor–receptor and receptor–signaling protein stoichiometries (149).

## Concluding Remarks

As our understanding of GPCRs has improved, revealing the concepts of ligand and receptor bias, allosterism, and oligomerization, so have biophysical technologies to monitor and evaluate their function in ever more physiologically relevant ways. There is now a bewildering array of approaches available, each with their own strengths and weaknesses. Rigorously controlled application of these technologies promises to unlock many more secrets of the ever important GPCR superfamily.

## Conflict of Interest Statement

In addition to being Head of Molecular Endocrinology and Pharmacology, Harry Perkins Institute of Medical Research and Centre for Medical Research, The University of Western Australia, Kevin D. G. Pfleger is a Chief Scientific Officer of Dimerix Bioscience, a spin-out company of The University of Western Australia that has been assigned the rights to the “Receptor-HIT.” Kevin D. G. Pfleger is an inventor on patents covering the technology and has a minor shareholding in Dimerix. Stephen J. Hill is a founding Director of the University of Nottingham spin-out company CellAura Technologies that markets fluorescent ligands. The other co-authors report no conflicts of interest.
